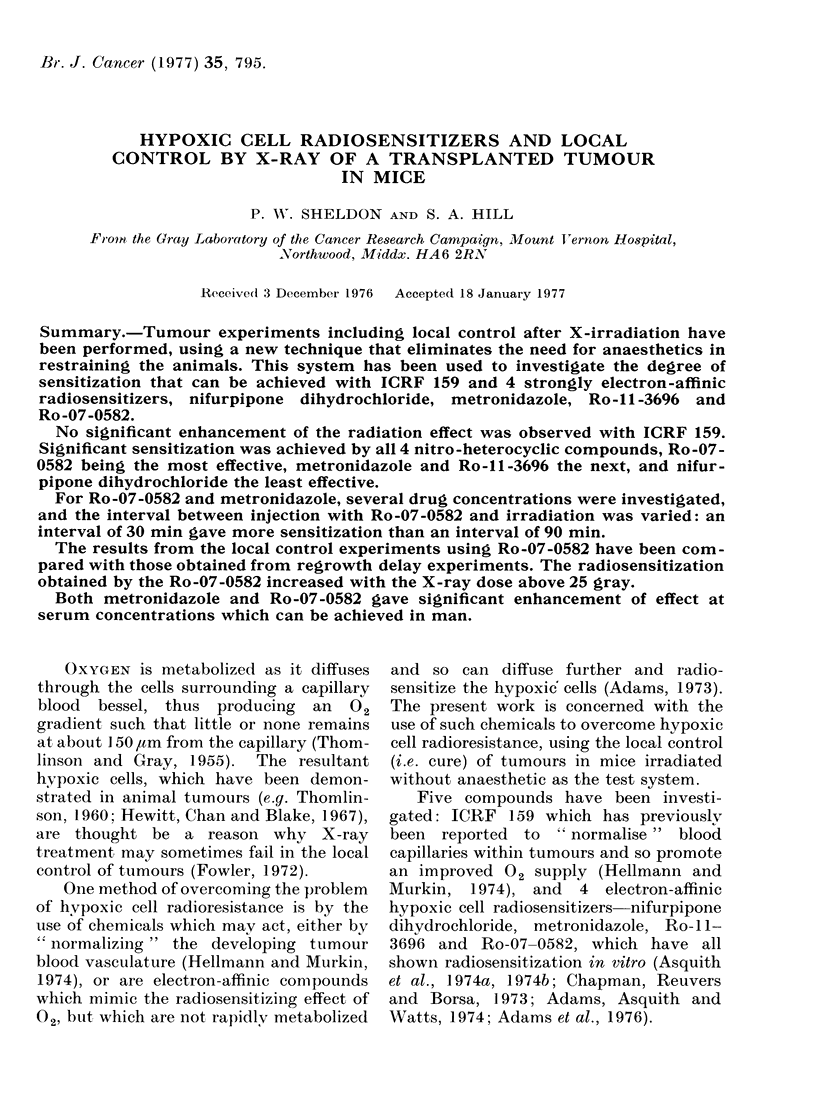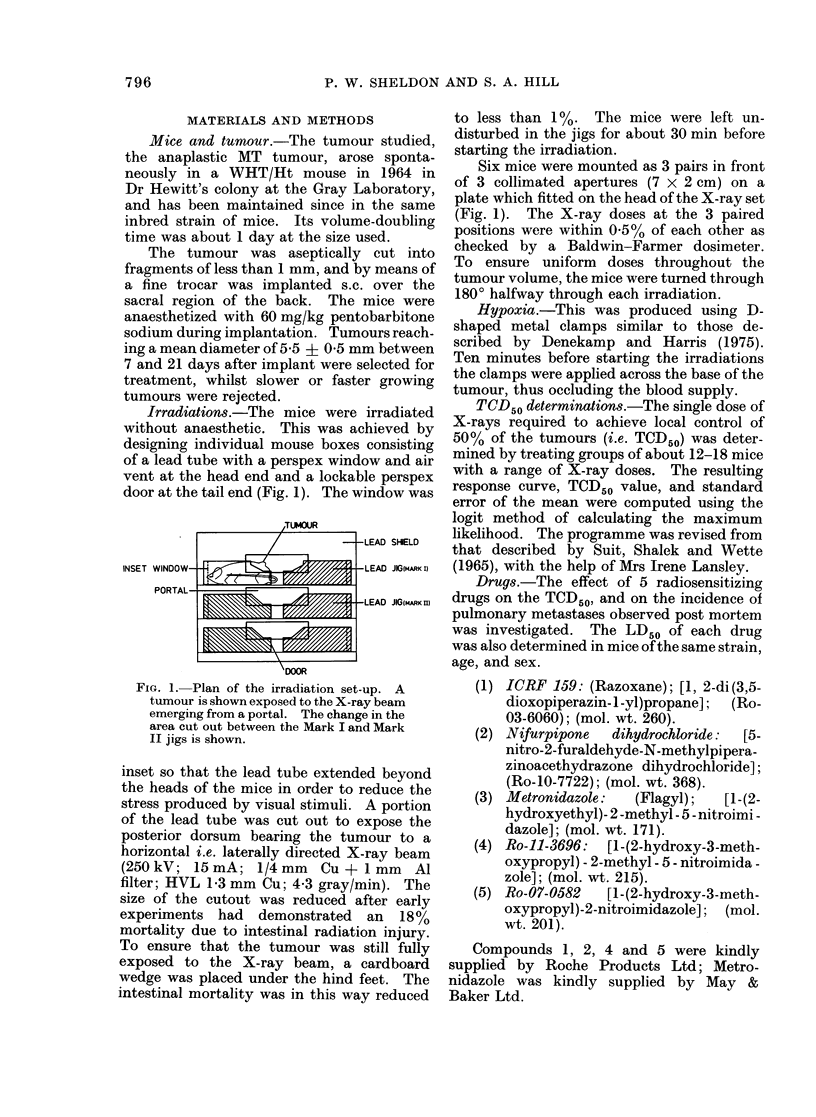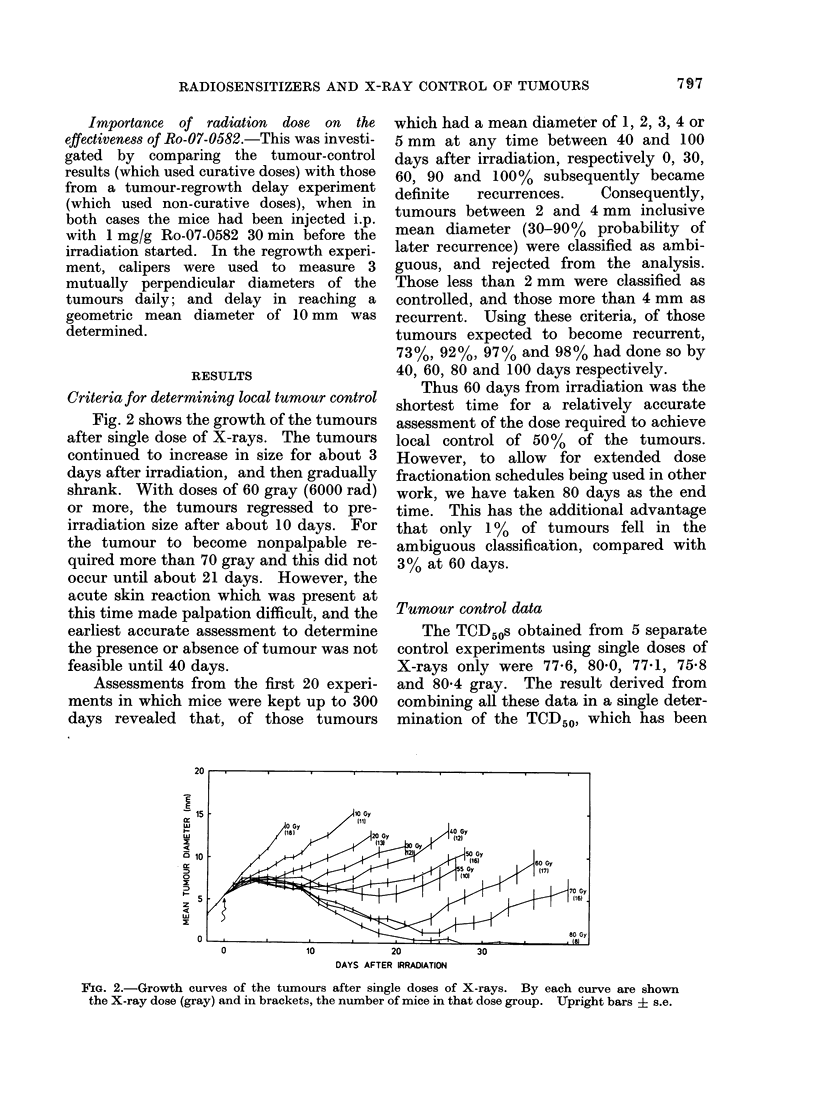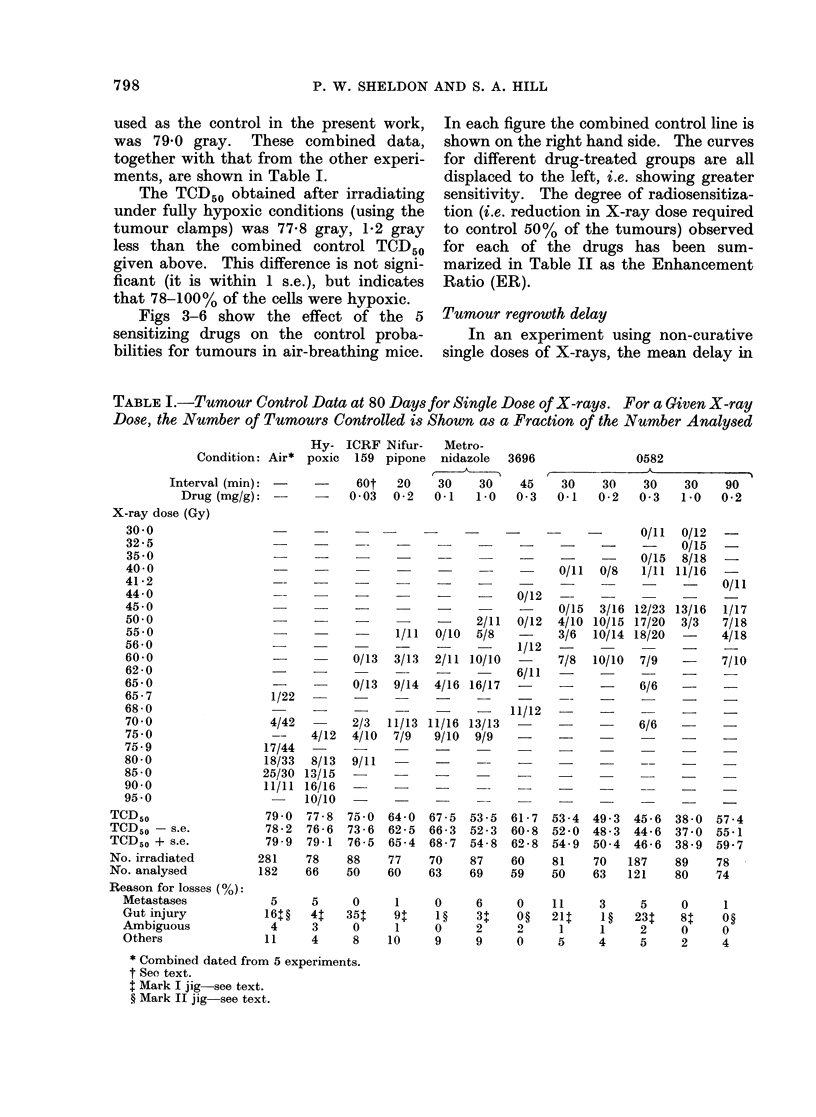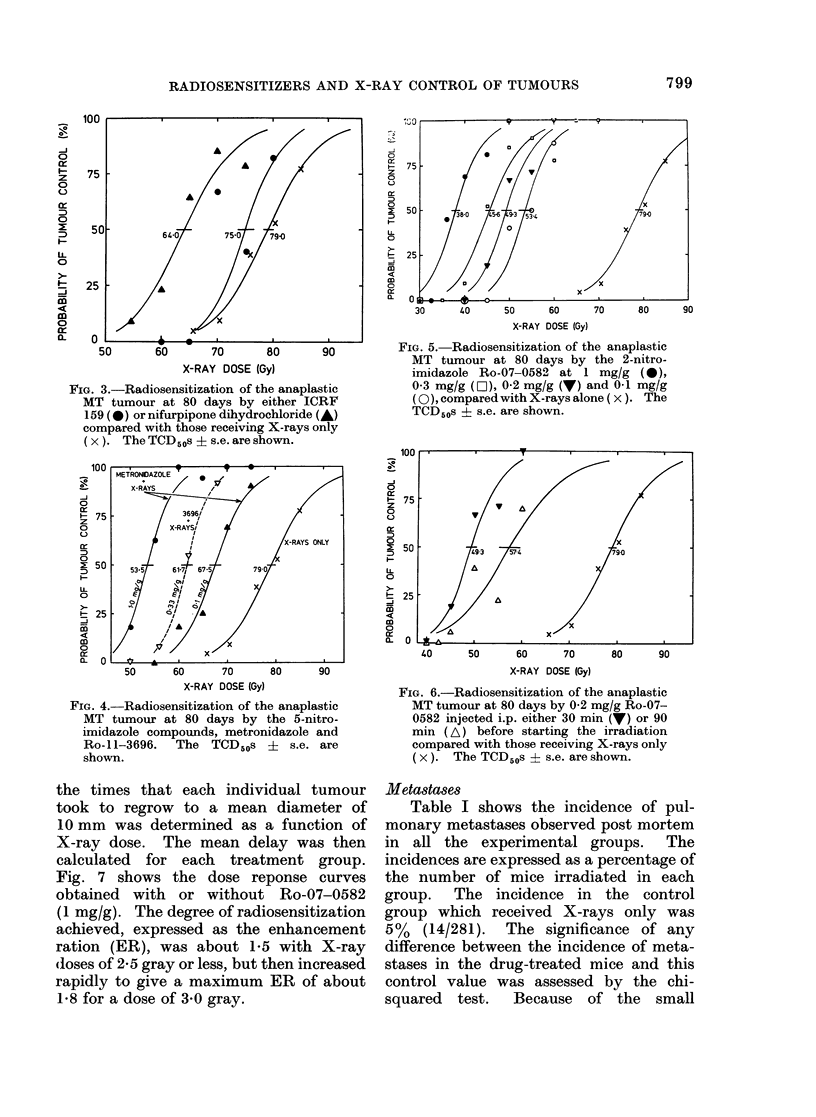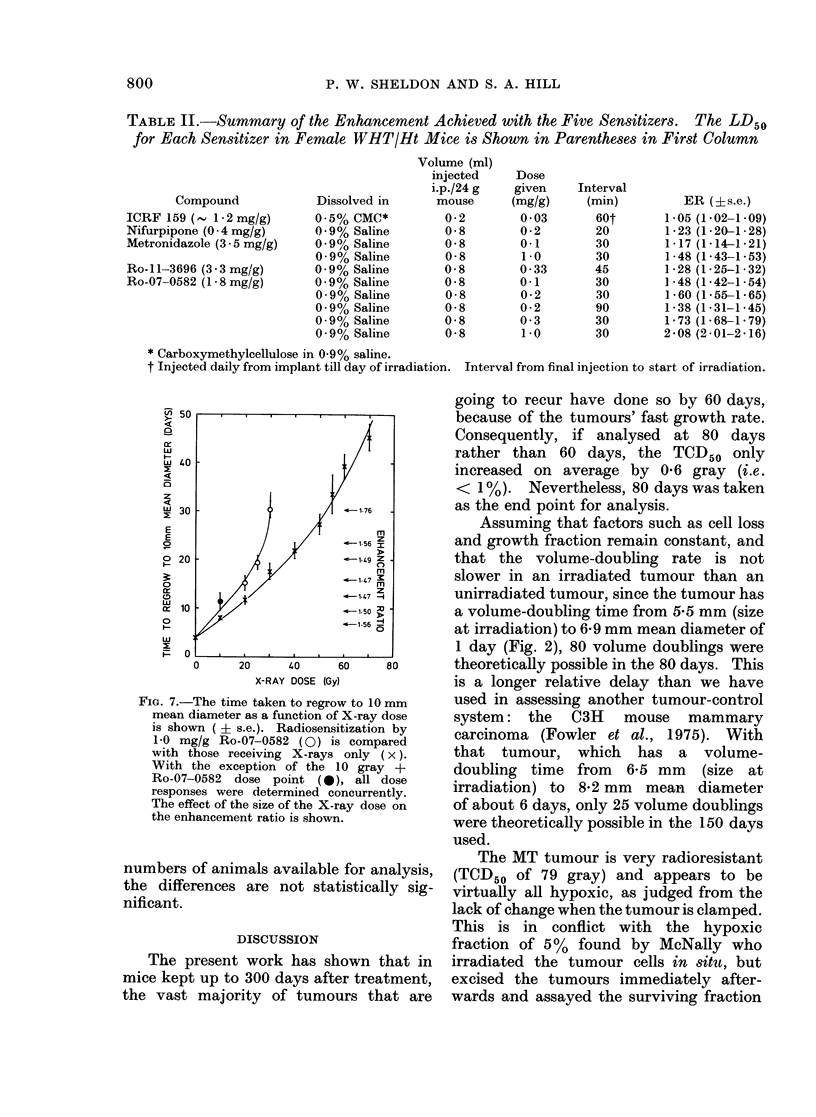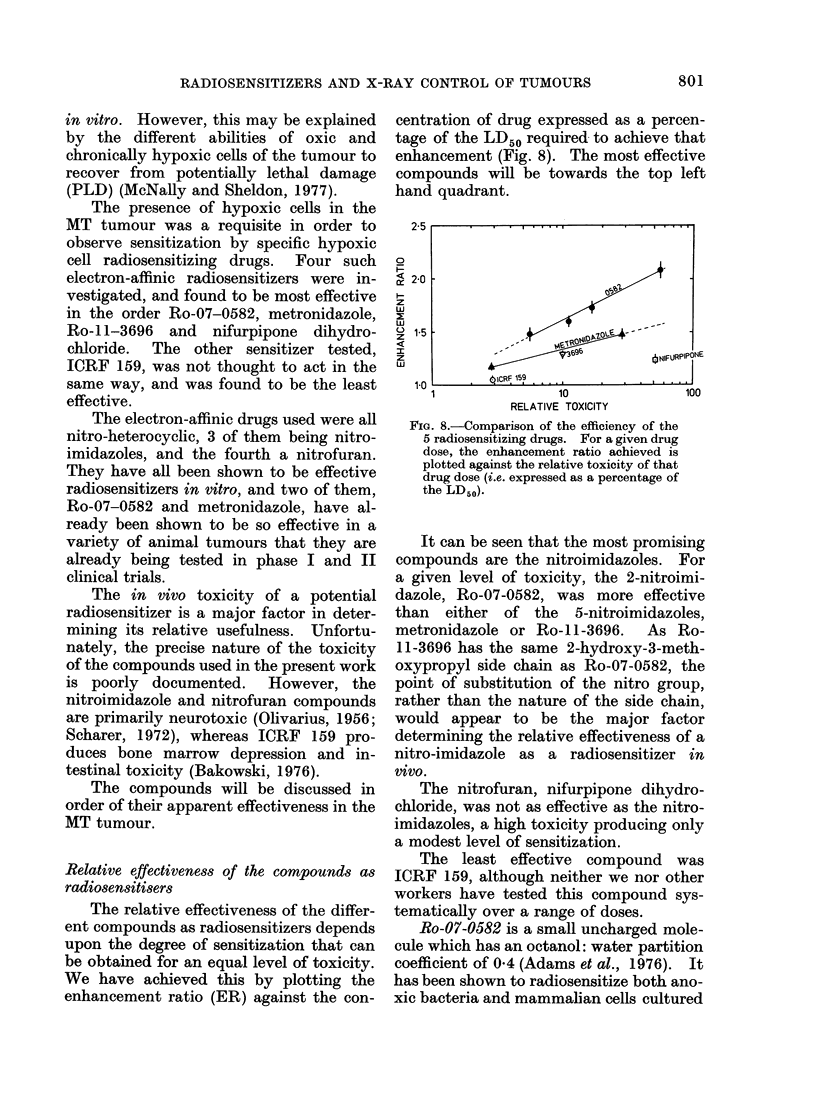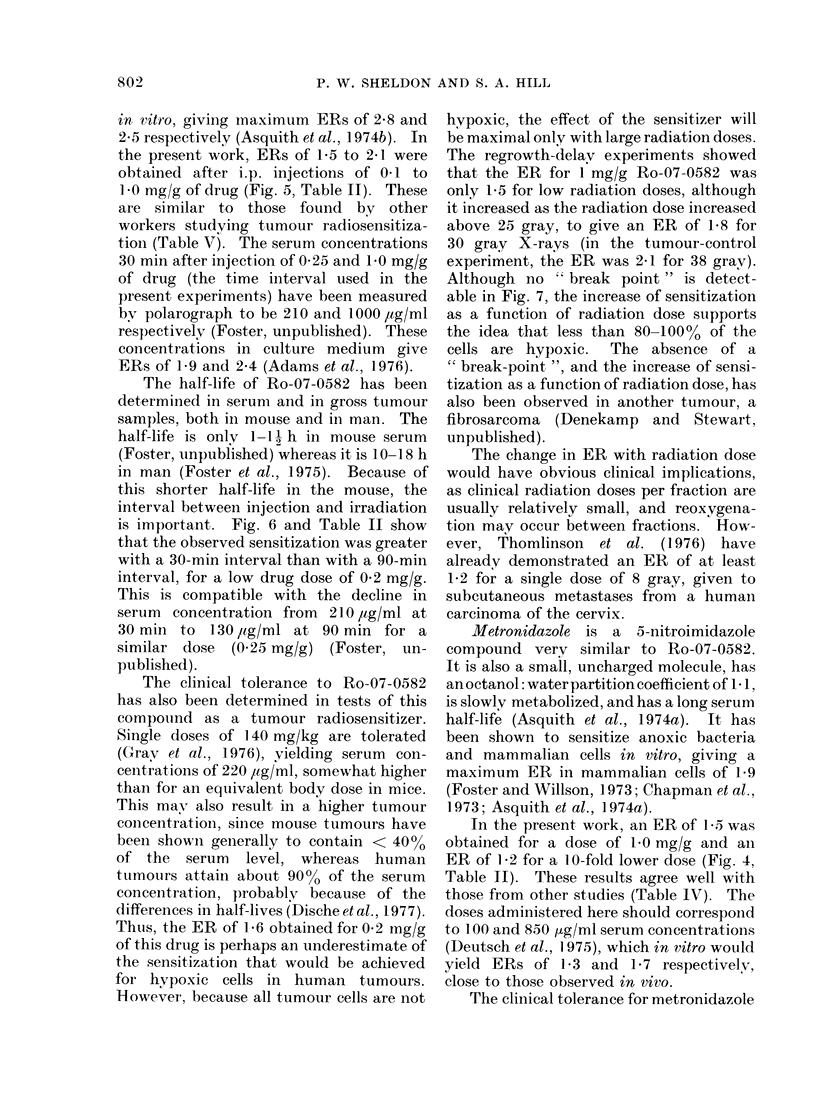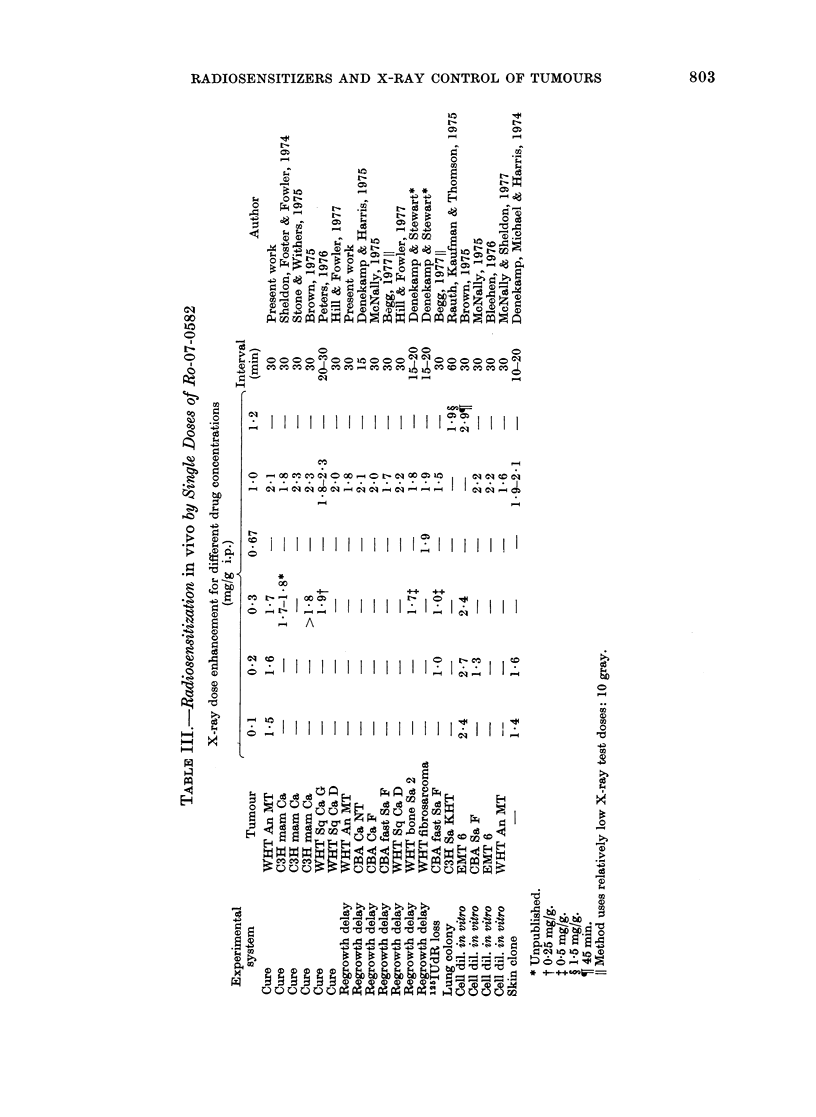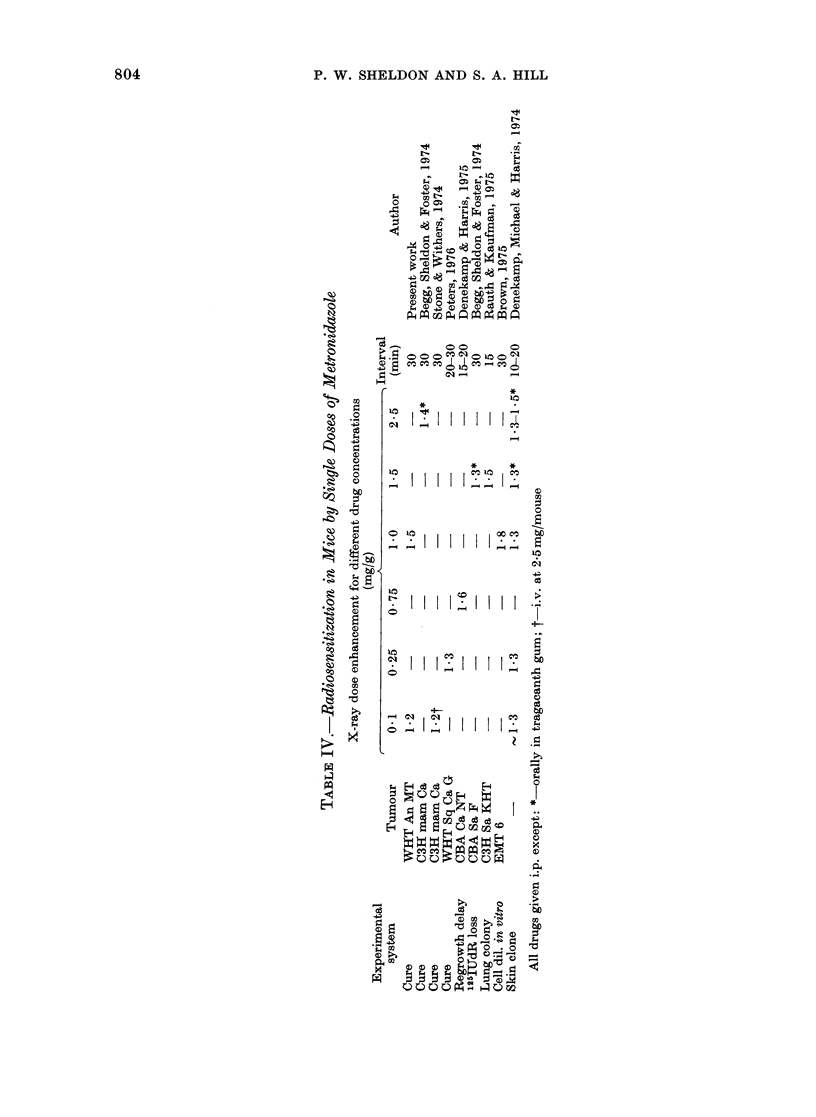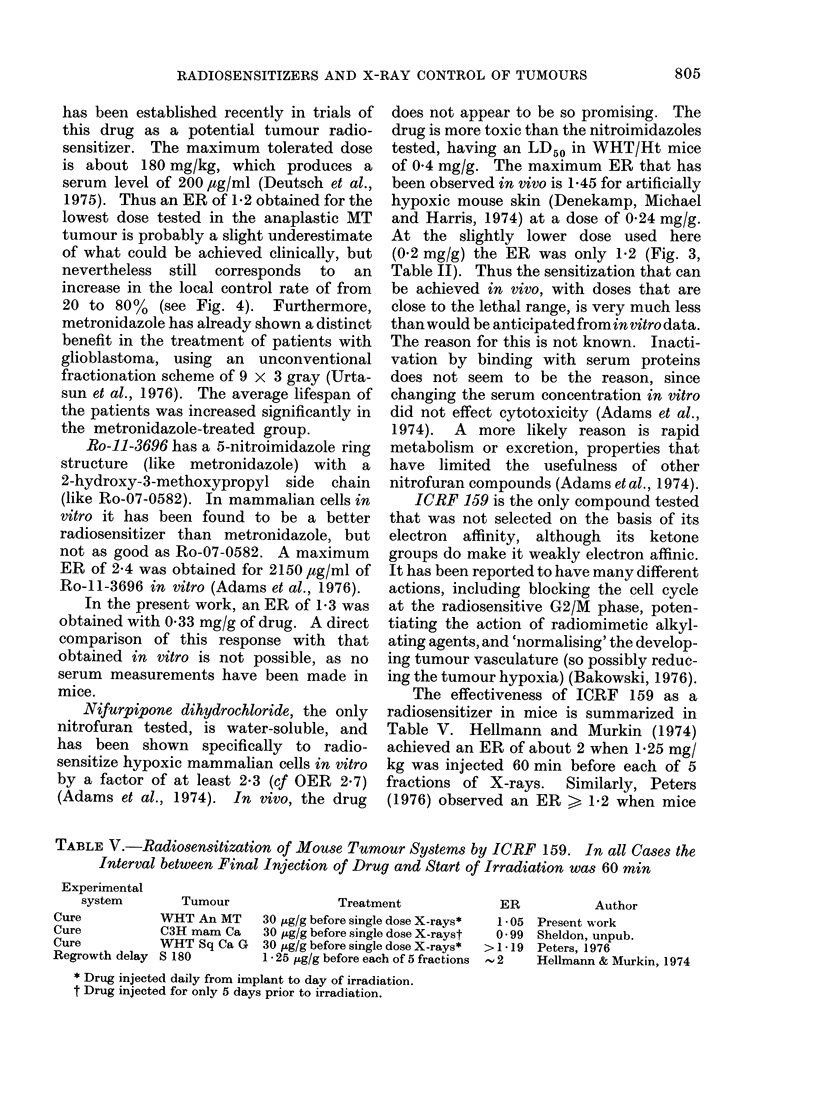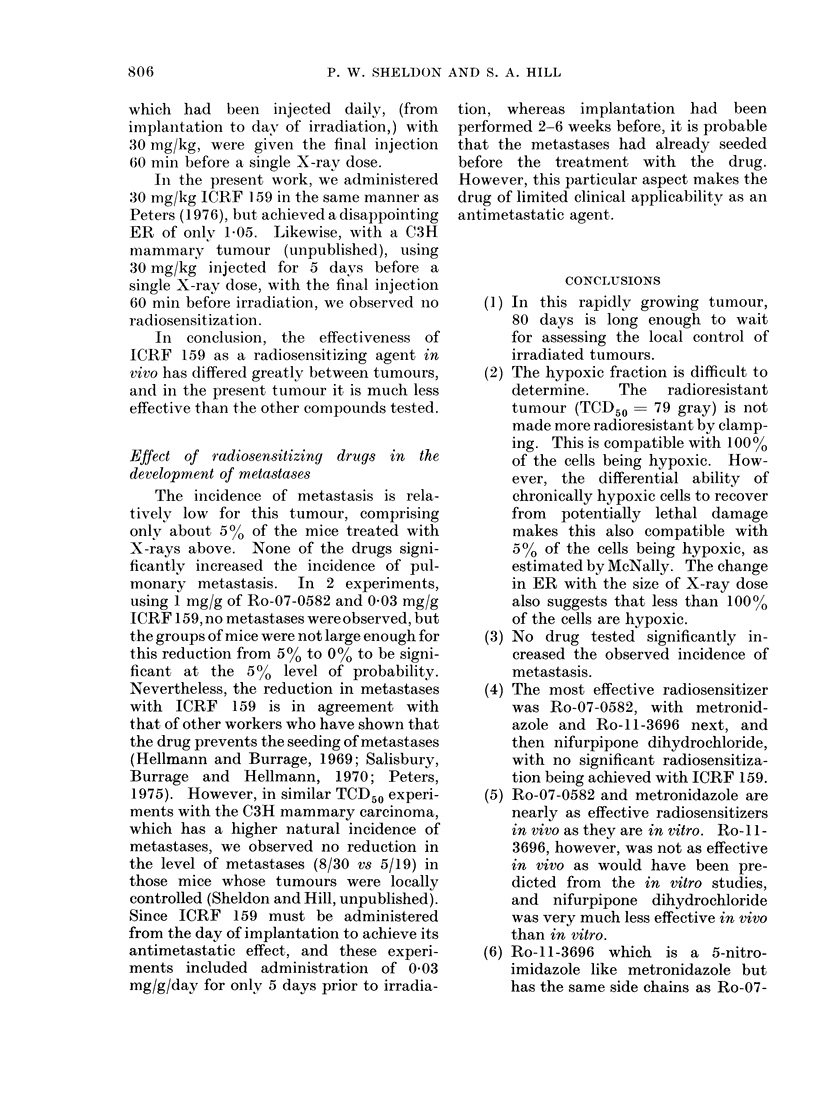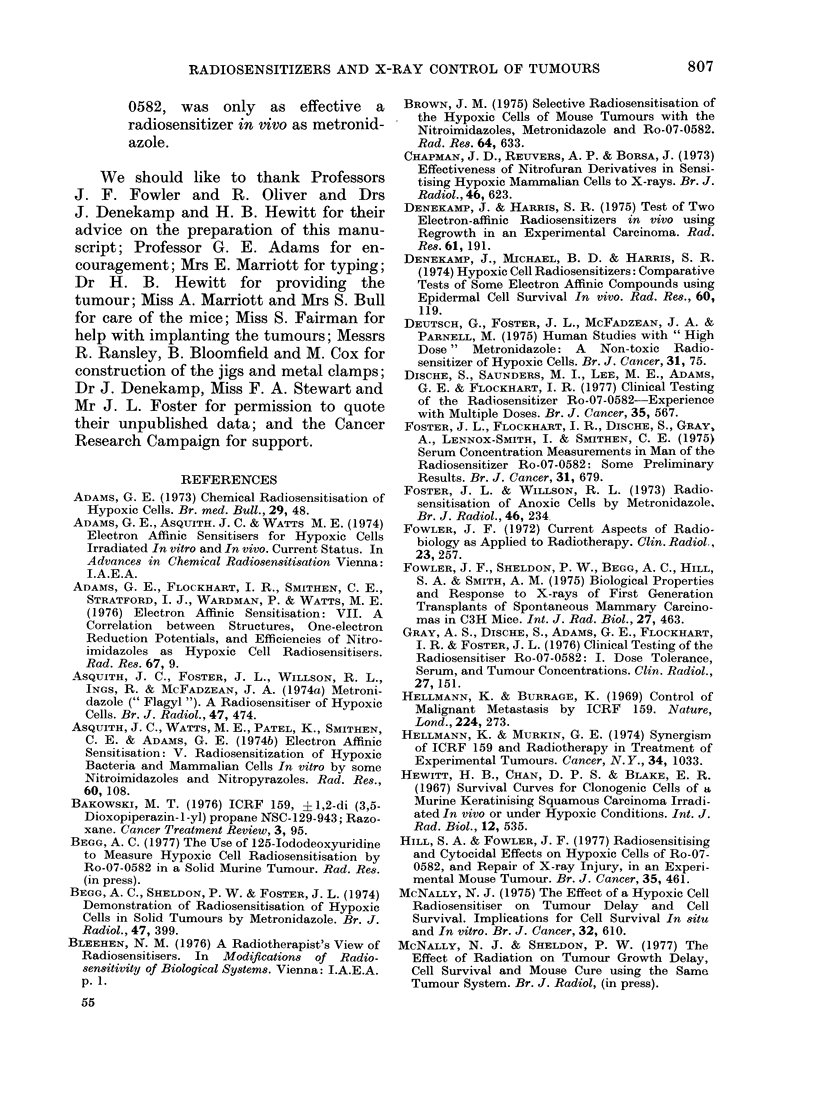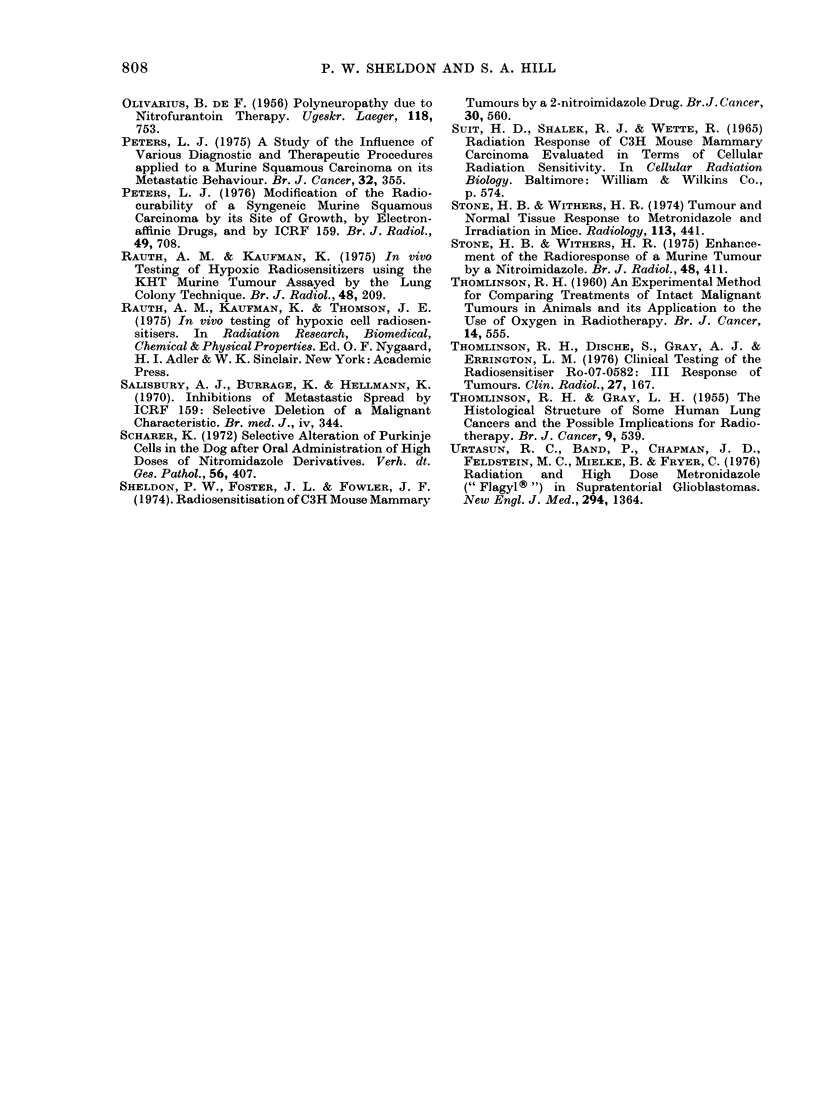# Hypoxic cell radiosensitizers and local control by X-ray of a transplanted tumour in mice.

**DOI:** 10.1038/bjc.1977.121

**Published:** 1977-06

**Authors:** P. W. Sheldon, S. A. Hill

## Abstract

Tumour experiments including local control after X-irradiation have been performed, using a new technique that eliminates the need for anaesthetics in restraining the animals. This system has been used to investigate the degree of sensitization that can be achieved with ICRF 159 and 4 strongly electron-affinic radiosensitizers, nifurpipone dihydrochloride, metronidazole, Ro-11-3696 and Ro-07-0582. No significant enhancement of the radiation effect was observed with ICRF 159. Significant sensitization was achieved by all 4 nitro-heterocyclic compoinds, Ro-07-0582 being the most effective, metronidazole and Ro-11-3696 the next, and nifurpipone dihydrochloride the least effective. For Ro-07-0582 and metronidazole, several concentrations were investigated, and the interval between injection with Ro-07-0582 and irradiation was varied: an interval of 30 min gave more sensitization than an interval of 90 min. The results from the local control experiments using Ro-07-0582 have been compared with those obtained from regrowth delay experiments. The radiosensitization obtained by the Ro-07-0582 increased with the X-ray dose above 25 gray. Both metronidazole and Ro-07-0582 gave significant enhancement effect at serum concentrations which can be achieved in man.


*Br. J. Cancer* (1977) **35**, 795.

### HYPOXIC CELL RADIOSENSITIZERS AND LOCAL CONTROL BY X-RAY OF A TRANSPLANTED TUMOUR IN MICE

P. W. SHELDON AND S. A. HILL

*From the Gray Laboratory of the Cancer Research Campaign, Mount Vernon Hospital, Northwood, Middx. HA6 2RN*

Received 3 December 1976 Accepted 18 January 1977

**Summary.—Tumour experiments including local control after X-irradiation have been performed, using a new technique that eliminates the need for anaesthetics in restraining the animals. This system has been used to investigate the degree of sensitization that can be achieved with ICRF 159 and 4 strongly electron-affinic radiosensitizers, nifurpipone dihydrochloride, metronidazole, Ro-11-3696 and Ro-07-0582.**

**No significant enhancement of the radiation effect was observed with ICRF 159. Significant sensitization was achieved by all 4 nitro-heterocyclic compounds, Ro-07-0582 being the most effective, metronidazole and Ro-11-3696 the next, and nifurpipone dihydrochloride the least effective.**

**For Ro-07-0582 and metronidazole, several drug concentrations were investigated, and the interval between injection with Ro-07-0582 and irradiation was varied: an interval of 30 min gave more sensitization than an interval of 90 min.**

**The results from the local control experiments using Ro-07-0582 have been compared with those obtained from regrowth delay experiments. The radiosensitization obtained by the Ro-07-0582 increased with the X-ray dose above 25 gray.**

**Both metronidazole and Ro-07-0582 gave significant enhancement of effect at serum concentrations which can be achieved in man.**

OXYGEN is metabolized as it diffuses through the cells surrounding a capillary blood bessel, thus producing an $O_2$ gradient such that little or none remains at about 150 $\mu$m from the capillary (Thomlinson and Gray, 1955). The resultant hypoxic cells, which have been demonstrated in animal tumours (*e.g.* Thomlinson, 1960; Hewitt, Chan and Blake, 1967), are thought be a reason why X-ray treatment may sometimes fail in the local control of tumours (Fowler, 1972).

One method of overcoming the problem of hypoxic cell radioresistance is by the use of chemicals which may act, either by "normalizing" the developing tumour blood vasculature (Hellmann and Murkin, 1974), or are electron-affinic compounds which mimic the radiosensitizing effect of $O_2$, but which are not rapidly metabolized and so can diffuse further and radiosensitize the hypoxic cells (Adams, 1973). The present work is concerned with the use of such chemicals to overcome hypoxic cell radioresistance, using the local control (*i.e.* cure) of tumours in mice irradiated without anaesthetic as the test system.

Five compounds have been investigated: ICRF 159 which has previously been reported to "normalise" blood capillaries within tumours and so promote an improved $O_2$ supply (Hellmann and Murkin, 1974), and 4 electron-affinic hypoxic cell radiosensitizers—nifurpipone dihydrochloride, metronidazole, Ro-11–3696 and Ro-07–0582, which have all shown radiosensitization *in vitro* (Asquith *et al.*, 1974*a*, 1974*b*; Chapman, Reuvers and Borsa, 1973; Adams, Asquith and Watts, 1974; Adams *et al.*, 1976).

MATERIALS AND METHODS

*Mice and tumour.*—The tumour studied, the anaplastic MT tumour, arose spontaneously in a WHT/Ht mouse in 1964 in Dr Hewitt's colony at the Gray Laboratory, and has been maintained since in the same inbred strain of mice. Its volume-doubling time was about 1 day at the size used.

The tumour was aseptically cut into fragments of less than 1 mm, and by means of a fine trocar was implanted s.c. over the sacral region of the back. The mice were anaesthetized with 60 mg/kg pentobarbitone sodium during implantation. Tumours reaching a mean diameter of 5·5 ± 0·5 mm between 7 and 21 days after implant were selected for treatment, whilst slower or faster growing tumours were rejected.

*Irradiations.*—The mice were irradiated without anaesthetic. This was achieved by designing individual mouse boxes consisting of a lead tube with a perspex window and air vent at the head end and a lockable perspex door at the tail end (Fig. 1). The window was inset so that the lead tube extended beyond the heads of the mice in order to reduce the stress produced by visual stimuli. A portion of the lead tube was cut out to expose the posterior dorsum bearing the tumour to a horizontal *i.e.* laterally directed X-ray beam (250 kV; 15 mA; 1/4 mm Cu + 1 mm Al filter; HVL 1·3 mm Cu; 4·3 gray/min). The size of the cutout was reduced after early experiments had demonstrated an 18% mortality due to intestinal radiation injury. To ensure that the tumour was still fully exposed to the X-ray beam, a cardboard wedge was placed under the hind feet. The intestinal mortality was in this way reduced to less than 1%. The mice were left undisturbed in the jigs for about 30 min before starting the irradiation.

FIG. 1.—Plan of the irradiation set-up. A tumour is shown exposed to the X-ray beam emerging from a portal. The change in the area cut out between the Mark I and Mark II jigs is shown.

Six mice were mounted as 3 pairs in front of 3 collimated apertures (7 × 2 cm) on a plate which fitted on the head of the X-ray set (Fig. 1). The X-ray doses at the 3 paired positions were within 0·5% of each other as checked by a Baldwin–Farmer dosimeter. To ensure uniform doses throughout the tumour volume, the mice were turned through 180° halfway through each irradiation.

*Hypoxia.*—This was produced using D-shaped metal clamps similar to those described by Denekamp and Harris (1975). Ten minutes before starting the irradiations the clamps were applied across the base of the tumour, thus occluding the blood supply.

*$TCD_{50}$ determinations.*—The single dose of X-rays required to achieve local control of 50% of the tumours (*i.e.* $TCD_{50}$) was determined by treating groups of about 12–18 mice with a range of X-ray doses. The resulting response curve, $TCD_{50}$ value, and standard error of the mean were computed using the logit method of calculating the maximum likelihood. The programme was revised from that described by Suit, Shalek and Wette (1965), with the help of Mrs Irene Lansley.

*Drugs.*—The effect of 5 radiosensitizing drugs on the $TCD_{50}$, and on the incidence of pulmonary metastases observed post mortem was investigated. The $LD_{50}$ of each drug was also determined in mice of the same strain, age, and sex.

(1) *ICRF 159:* (Razoxane); [1, 2-di(3,5-dioxopiperazin-1-yl)propane]; (Ro-03-6060); (mol. wt. 260).
(2) *Nifurpipone dihydrochloride:* [5-nitro-2-furaldehyde-N-methylpiperazinoacethydrazone dihydrochloride]; (Ro-10-7722); (mol. wt. 368).
(3) *Metronidazole:* (Flagyl); [1-(2-hydroxyethyl)-2-methyl-5-nitroimidazole]; (mol. wt. 171).
(4) *Ro-11-3696:* [1-(2-hydroxy-3-methoxypropyl)-2-methyl-5-nitroimidazole]; (mol. wt. 215).
(5) *Ro-07-0582* [1-(2-hydroxy-3-methoxypropyl)-2-nitroimidazole]; (mol. wt. 201).

Compounds 1, 2, 4 and 5 were kindly supplied by Roche Products Ltd; Metronidazole was kindly supplied by May & Baker Ltd.

*Importance of radiation dose on the effectiveness of Ro-07-0582.*—This was investigated by comparing the tumour-control results (which used curative doses) with those from a tumour-regrowth delay experiment (which used non-curative doses), when in both cases the mice had been injected i.p. with 1 mg/g Ro-07-0582 30 min before the irradiation started. In the regrowth experiment, calipers were used to measure 3 mutually perpendicular diameters of the tumours daily; and delay in reaching a geometric mean diameter of 10 mm was determined.

### RESULTS

### *Criteria for determining local tumour control*

Fig. 2 shows the growth of the tumours after single dose of X-rays. The tumours continued to increase in size for about 3 days after irradiation, and then gradually shrank. With doses of 60 gray (6000 rad) or more, the tumours regressed to pre-irradiation size after about 10 days. For the tumour to become nonpalpable required more than 70 gray and this did not occur until about 21 days. However, the acute skin reaction which was present at this time made palpation difficult, and the earliest accurate assessment to determine the presence or absence of tumour was not feasible until 40 days.

Assessments from the first 20 experiments in which mice were kept up to 300 days revealed that, of those tumours which had a mean diameter of 1, 2, 3, 4 or 5 mm at any time between 40 and 100 days after irradiation, respectively 0, 30, 60, 90 and 100% subsequently became definite recurrences. Consequently, tumours between 2 and 4 mm inclusive mean diameter (30–90% probability of later recurrence) were classified as ambiguous, and rejected from the analysis. Those less than 2 mm were classified as controlled, and those more than 4 mm as recurrent. Using these criteria, of those tumours expected to become recurrent, 73%, 92%, 97% and 98% had done so by 40, 60, 80 and 100 days respectively.

Thus 60 days from irradiation was the shortest time for a relatively accurate assessment of the dose required to achieve local control of 50% of the tumours. However, to allow for extended dose fractionation schedules being used in other work, we have taken 80 days as the end time. This has the additional advantage that only 1% of tumours fell in the ambiguous classification, compared with 3% at 60 days.

### *Tumour control data*

The $TCD_{50}$s obtained from 5 separate control experiments using single doses of X-rays only were 77·6, 80·0, 77·1, 75·8 and 80·4 gray. The result derived from combining all these data in a single determination of the $TCD_{50}$, which has been

FIG. 2.—Growth curves of the tumours after single doses of X-rays. By each curve are shown the X-ray dose (gray) and in brackets, the number of mice in that dose group. Upright bars ± s.e.

used as the control in the present work, was 79·0 gray. These combined data, together with that from the other experiments, are shown in Table I.

The $TCD_{50}$ obtained after irradiating under fully hypoxic conditions (using the tumour clamps) was 77·8 gray, 1·2 gray less than the combined control $TCD_{50}$ given above. This difference is not significant (it is within 1 s.e.), but indicates that 78–100% of the cells were hypoxic.

Figs 3–6 show the effect of the 5 sensitizing drugs on the control probabilities for tumours in air-breathing mice. In each figure the combined control line is shown on the right hand side. The curves for different drug-treated groups are all displaced to the left, *i.e.* showing greater sensitivity. The degree of radiosensitization (*i.e.* reduction in X-ray dose required to control 50% of the tumours) observed for each of the drugs has been summarized in Table II as the Enhancement Ratio (ER).

*Tumour regrowth delay*

In an experiment using non-curative single doses of X-rays, the mean delay in

TABLE I.—*Tumour Control Data at* 80 *Days for Single Dose of X-rays. For a Given X-ray Dose, the Number of Tumours Controlled is Shown as a Fraction of the Number Analysed*

| Condition: | Air* | Hypoxic | ICRF 159 | Nifurpipone | Metronidazole | | 3696 | 0582 | | | | |
|---|---|---|---|---|---|---|---|---|---|---|---|---|
| Interval (min): | — | — | 60† | 20 | 30 | 30 | 45 | 30 | 30 | 30 | 30 | 90 |
| Drug (mg/g): | — | — | 0·03 | 0·2 | 0·1 | 1·0 | 0·3 | 0·1 | 0·2 | 0·3 | 1·0 | 0·2 |
| X-ray dose (Gy) | | | | | | | | | | | | |
| 30·0 | — | — | — | — | — | — | — | — | — | 0/11 | 0/12 | — |
| 32·5 | — | — | — | — | — | — | — | — | — | — | 0/15 | — |
| 35·0 | — | — | — | — | — | — | — | — | — | 0/15 | 8/18 | — |
| 40·0 | — | — | — | — | — | — | — | 0/11 | 0/8 | 1/11 | 11/16 | — |
| 41·2 | — | — | — | — | — | — | — | — | — | — | — | 0/11 |
| 44·0 | — | — | — | — | — | — | 0/12 | — | — | — | — | — |
| 45·0 | — | — | — | — | — | — | — | 0/15 | 3/16 | 12/23 | 13/16 | 1/17 |
| 50·0 | — | — | — | — | — | 2/11 | 0/12 | 4/10 | 10/15 | 17/20 | 3/3 | 7/18 |
| 55·0 | — | — | — | 1/11 | 0/10 | 5/8 | — | 3/6 | 10/14 | 18/20 | — | 4/18 |
| 56·0 | — | — | — | — | — | — | 1/12 | — | — | — | — | — |
| 60·0 | — | — | 0/13 | 3/13 | 2/11 | 10/10 | — | 7/8 | 10/10 | 7/9 | — | 7/10 |
| 62·0 | — | — | — | — | — | — | 6/11 | — | — | — | — | — |
| 65·0 | — | — | 0/13 | 9/14 | 4/16 | 16/17 | — | — | — | 6/6 | — | — |
| 65·7 | 1/22 | — | — | — | — | — | — | — | — | — | — | — |
| 68·0 | — | — | — | — | — | — | 11/12 | — | — | — | — | — |
| 70·0 | 4/42 | — | 2/3 | 11/13 | 11/16 | 13/13 | — | — | — | 6/6 | — | — |
| 75·0 | — | 4/12 | 4/10 | 7/9 | 9/10 | 9/9 | — | — | — | — | — | — |
| 75·9 | 17/44 | — | — | — | — | — | — | — | — | — | — | — |
| 80·0 | 18/33 | 8/13 | 9/11 | — | — | — | — | — | — | — | — | — |
| 85·0 | 25/30 | 13/15 | — | — | — | — | — | — | — | — | — | — |
| 90·0 | 11/11 | 16/16 | — | — | — | — | — | — | — | — | — | — |
| 95·0 | — | 10/10 | — | — | — | — | — | — | — | — | — | — |
| $TCD_{50}$ | 79·0 | 77·8 | 75·0 | 64·0 | 67·5 | 53·5 | 61·7 | 53·4 | 49·3 | 45·6 | 38·0 | 57·4 |
| $TCD_{50}$ − s.e. | 78·2 | 76·6 | 73·6 | 62·5 | 66·3 | 52·3 | 60·8 | 52·0 | 48·3 | 44·6 | 37·0 | 55·1 |
| $TCD_{50}$ + s.e. | 79·9 | 79·1 | 76·5 | 65·4 | 68·7 | 54·8 | 62·8 | 54·9 | 50·4 | 46·6 | 38·9 | 59·7 |
| No. irradiated | 281 | 78 | 88 | 77 | 70 | 87 | 60 | 81 | 70 | 187 | 89 | 78 |
| No. analysed | 182 | 66 | 50 | 60 | 63 | 69 | 59 | 50 | 63 | 121 | 80 | 74 |
| Reason for losses (%): | | | | | | | | | | | | |
| Metastases | 5 | 5 | 0 | 1 | 0 | 6 | 0 | 11 | 3 | 5 | 0 | 1 |
| Gut injury | 16‡§ | 4‡ | 35‡ | 9‡ | 1§ | 3‡ | 0§ | 21‡ | 1§ | 23‡ | 8‡ | 0§ |
| Ambiguous | 4 | 3 | 0 | 1 | 0 | 2 | 2 | 1 | 1 | 2 | 0 | 0 |
| Others | 11 | 4 | 8 | 10 | 9 | 9 | 0 | 5 | 4 | 5 | 2 | 4 |

* Combined dated from 5 experiments.
† See text.
‡ Mark I jig—see text.
§ Mark II jig—see text.

FIG. 3.—Radiosensitization of the anaplastic MT tumour at 80 days by either ICRF 159 (●) or nifurpipone dihydrochloride (▲) compared with those receiving X-rays only (×). The $TCD_{50}$s ± s.e. are shown.

FIG. 4.—Radiosensitization of the anaplastic MT tumour at 80 days by the 5-nitroimidazole compounds, metronidazole and Ro-11-3696. The $TCD_{50}$s ± s.e. are shown.

FIG. 5.—Radiosensitization of the anaplastic MT tumour at 80 days by the 2-nitroimidazole Ro-07-0582 at 1 mg/g (●), 0·3 mg/g (□), 0·2 mg/g (▼) and 0·1 mg/g (○), compared with X-rays alone (×). The $TCD_{50}$s ± s.e. are shown.

FIG. 6.—Radiosensitization of the anaplastic MT tumour at 80 days by 0·2 mg/g Ro-07-0582 injected i.p. either 30 min (▼) or 90 min (△) before starting the irradiation compared with those receiving X-rays only (×). The $TCD_{50}$s ± s.e. are shown.

the times that each individual tumour took to regrow to a mean diameter of 10 mm was determined as a function of X-ray dose. The mean delay was then calculated for each treatment group. Fig. 7 shows the dose reponse curves obtained with or without Ro-07-0582 (1 mg/g). The degree of radiosensitization achieved, expressed as the enhancement ration (ER), was about 1·5 with X-ray doses of 2·5 gray or less, but then increased rapidly to give a maximum ER of about 1·8 for a dose of 3·0 gray.

*Metastases*

Table I shows the incidence of pulmonary metastases observed post mortem in all the experimental groups. The incidences are expressed as a percentage of the number of mice irradiated in each group. The incidence in the control group which received X-rays only was 5% (14/281). The significance of any difference between the incidence of metastases in the drug-treated mice and this control value was assessed by the chi-squared test. Because of the small

TABLE II.—*Summary of the Enhancement Achieved with the Five Sensitizers. The $LD_{50}$ for Each Sensitizer in Female WHT/Ht Mice is Shown in Parentheses in First Column*

| Compound | Dissolved in | Volume (ml) injected i.p./24 g mouse | Dose given (mg/g) | Interval (min) | ER (±s.e.) |
|---|---|---|---|---|---|
| ICRF 159 (~ 1·2 mg/g) | 0·5% CMC* | 0·2 | 0·03 | 60† | 1·05 (1·02–1·09) |
| Nifurpipone (0·4 mg/g) | 0·9% Saline | 0·8 | 0·2 | 20 | 1·23 (1·20–1·28) |
| Metronidazole (3·5 mg/g) | 0·9% Saline | 0·8 | 0·1 | 30 | 1·17 (1·14–1·21) |
| | 0·9% Saline | 0·8 | 1·0 | 30 | 1·48 (1·43–1·53) |
| Ro-11–3696 (3·3 mg/g) | 0·9% Saline | 0·8 | 0·33 | 45 | 1·28 (1·25–1·32) |
| Ro-07–0582 (1·8 mg/g) | 0·9% Saline | 0·8 | 0·1 | 30 | 1·48 (1·42–1·54) |
| | 0·9% Saline | 0·8 | 0·2 | 30 | 1·60 (1·55–1·65) |
| | 0·9% Saline | 0·8 | 0·2 | 90 | 1·38 (1·31–1·45) |
| | 0·9% Saline | 0·8 | 0·3 | 30 | 1·73 (1·68–1·79) |
| | 0·9% Saline | 0·8 | 1·0 | 30 | 2·08 (2·01–2·16) |

* Carboxymethylcellulose in 0·9% saline.
† Injected daily from implant till day of irradiation. Interval from final injection to start of irradiation.

FIG. 7.—The time taken to regrow to 10 mm mean diameter as a function of X-ray dose is shown (± s.e.). Radiosensitization by 1·0 mg/g Ro-07–0582 (○) is compared with those receiving X-rays only (×). With the exception of the 10 gray + Ro-07–0582 dose point (●), all dose responses were determined concurrently. The effect of the size of the X-ray dose on the enhancement ratio is shown.

numbers of animals available for analysis, the differences are not statistically significant.

DISCUSSION

The present work has shown that in mice kept up to 300 days after treatment, the vast majority of tumours that are going to recur have done so by 60 days, because of the tumours' fast growth rate. Consequently, if analysed at 80 days rather than 60 days, the $TCD_{50}$ only increased on average by 0·6 gray (*i.e.* < 1%). Nevertheless, 80 days was taken as the end point for analysis.

Assuming that factors such as cell loss and growth fraction remain constant, and that the volume-doubling rate is not slower in an irradiated tumour than an unirradiated tumour, since the tumour has a volume-doubling time from 5·5 mm (size at irradiation) to 6·9 mm mean diameter of 1 day (Fig. 2), 80 volume doublings were theoretically possible in the 80 days. This is a longer relative delay than we have used in assessing another tumour-control system: the C3H mouse mammary carcinoma (Fowler *et al.*, 1975). With that tumour, which has a volume-doubling time from 6·5 mm (size at irradiation) to 8·2 mm mean diameter of about 6 days, only 25 volume doublings were theoretically possible in the 150 days used.

The MT tumour is very radioresistant ($TCD_{50}$ of 79 gray) and appears to be virtually all hypoxic, as judged from the lack of change when the tumour is clamped. This is in conflict with the hypoxic fraction of 5% found by McNally who irradiated the tumour cells *in situ*, but excised the tumours immediately afterwards and assayed the surviving fraction

*in vitro*. However, this may be explained by the different abilities of oxic and chronically hypoxic cells of the tumour to recover from potentially lethal damage (PLD) (McNally and Sheldon, 1977).

The presence of hypoxic cells in the MT tumour was a requisite in order to observe sensitization by specific hypoxic cell radiosensitizing drugs. Four such electron-affinic radiosensitizers were investigated, and found to be most effective in the order Ro-07–0582, metronidazole, Ro-11–3696 and nifurpipone dihydrochloride. The other sensitizer tested, ICRF 159, was not thought to act in the same way, and was found to be the least effective.

The electron-affinic drugs used were all nitro-heterocyclic, 3 of them being nitroimidazoles, and the fourth a nitrofuran. They have all been shown to be effective radiosensitizers *in vitro*, and two of them, Ro-07–0582 and metronidazole, have already been shown to be so effective in a variety of animal tumours that they are already being tested in phase I and II clinical trials.

The *in vivo* toxicity of a potential radiosensitizer is a major factor in determining its relative usefulness. Unfortunately, the precise nature of the toxicity of the compounds used in the present work is poorly documented. However, the nitroimidazole and nitrofuran compounds are primarily neurotoxic (Olivarius, 1956; Scharer, 1972), whereas ICRF 159 produces bone marrow depression and intestinal toxicity (Bakowski, 1976).

The compounds will be discussed in order of their apparent effectiveness in the MT tumour.

*Relative effectiveness of the compounds as radiosensitisers*

The relative effectiveness of the different compounds as radiosensitizers depends upon the degree of sensitization that can be obtained for an equal level of toxicity. We have achieved this by plotting the enhancement ratio (ER) against the concentration of drug expressed as a percentage of the $LD_{50}$ required to achieve that enhancement (Fig. 8). The most effective compounds will be towards the top left hand quadrant.

FIG. 8.—Comparison of the efficiency of the 5 radiosensitizing drugs. For a given drug dose, the enhancement ratio achieved is plotted against the relative toxicity of that drug dose (*i.e.* expressed as a percentage of the $LD_{50}$).

It can be seen that the most promising compounds are the nitroimidazoles. For a given level of toxicity, the 2-nitroimidazole, Ro-07-0582, was more effective than either of the 5-nitroimidazoles, metronidazole or Ro-11-3696. As Ro-11-3696 has the same 2-hydroxy-3-methoxypropyl side chain as Ro-07-0582, the point of substitution of the nitro group, rather than the nature of the side chain, would appear to be the major factor determining the relative effectiveness of a nitro-imidazole as a radiosensitizer *in vivo*.

The nitrofuran, nifurpipone dihydrochloride, was not as effective as the nitroimidazoles, a high toxicity producing only a modest level of sensitization.

The least effective compound was ICRF 159, although neither we nor other workers have tested this compound systematically over a range of doses.

*Ro-07-0582* is a small uncharged molecule which has an octanol: water partition coefficient of 0·4 (Adams *et al.*, 1976). It has been shown to radiosensitize both anoxic bacteria and mammalian cells cultured

*in vitro*, giving maximum ERs of 2·8 and 2·5 respectively (Asquith *et al.*, 1974*b*). In the present work, ERs of 1·5 to 2·1 were obtained after i.p. injections of 0·1 to 1·0 mg/g of drug (Fig. 5, Table II). These are similar to those found by other workers studying tumour radiosensitization (Table V). The serum concentrations 30 min after injection of 0·25 and 1·0 mg/g of drug (the time interval used in the present experiments) have been measured by polarograph to be 210 and 1000 $\mu$g/ml respectively (Foster, unpublished). These concentrations in culture medium give ERs of 1·9 and 2·4 (Adams *et al.*, 1976).

The half-life of Ro-07-0582 has been determined in serum and in gross tumour samples, both in mouse and in man. The half-life is only 1–1½ h in mouse serum (Foster, unpublished) whereas it is 10–18 h in man (Foster *et al.*, 1975). Because of this shorter half-life in the mouse, the interval between injection and irradiation is important. Fig. 6 and Table II show that the observed sensitization was greater with a 30-min interval than with a 90-min interval, for a low drug dose of 0·2 mg/g. This is compatible with the decline in serum concentration from 210 $\mu$g/ml at 30 min to 130 $\mu$g/ml at 90 min for a similar dose (0·25 mg/g) (Foster, unpublished).

The clinical tolerance to Ro-07-0582 has also been determined in tests of this compound as a tumour radiosensitizer. Single doses of 140 mg/kg are tolerated (Gray *et al.*, 1976), yielding serum concentrations of 220 $\mu$g/ml, somewhat higher than for an equivalent body dose in mice. This may also result in a higher tumour concentration, since mouse tumours have been shown generally to contain $< 40\%$ of the serum level, whereas human tumours attain about 90% of the serum concentration, probably because of the differences in half-lives (Dische *et al.*, 1977). Thus, the ER of 1·6 obtained for 0·2 mg/g of this drug is perhaps an underestimate of the sensitization that would be achieved for hypoxic cells in human tumours. However, because all tumour cells are not hypoxic, the effect of the sensitizer will be maximal only with large radiation doses. The regrowth-delay experiments showed that the ER for 1 mg/g Ro-07-0582 was only 1·5 for low radiation doses, although it increased as the radiation dose increased above 25 gray, to give an ER of 1·8 for 30 gray X-rays (in the tumour-control experiment, the ER was 2·1 for 38 gray). Although no "break point" is detectable in Fig. 7, the increase of sensitization as a function of radiation dose supports the idea that less than 80–100% of the cells are hypoxic. The absence of a "break-point", and the increase of sensitization as a function of radiation dose, has also been observed in another tumour, a fibrosarcoma (Denekamp and Stewart, unpublished).

The change in ER with radiation dose would have obvious clinical implications, as clinical radiation doses per fraction are usually relatively small, and reoxygenation may occur between fractions. However, Thomlinson *et al.* (1976) have already demonstrated an ER of at least 1·2 for a single dose of 8 gray, given to subcutaneous metastases from a human carcinoma of the cervix.

*Metronidazole* is a 5-nitroimidazole compound very similar to Ro-07-0582. It is also a small, uncharged molecule, has an octanol : water partition coefficient of 1·1, is slowly metabolized, and has a long serum half-life (Asquith *et al.*, 1974*a*). It has been shown to sensitize anoxic bacteria and mammalian cells *in vitro*, giving a maximum ER in mammalian cells of 1·9 (Foster and Willson, 1973; Chapman *et al.*, 1973; Asquith *et al.*, 1974*a*).

In the present work, an ER of 1·5 was obtained for a dose of 1·0 mg/g and an ER of 1·2 for a 10-fold lower dose (Fig. 4, Table II). These results agree well with those from other studies (Table IV). The doses administered here should correspond to 100 and 850 $\mu$g/ml serum concentrations (Deutsch *et al.*, 1975), which *in vitro* would yield ERs of 1·3 and 1·7 respectively, close to those observed *in vivo*.

The clinical tolerance for metronidazole

TABLE III.—*Radiosensitization* in vivo *by Single Doses of Ro*-07-0582

| Experimental system | Tumour | X-ray dose enhancement for different drug concentrations (mg/g i.p.) 0·1 | 0·2 | 0·3 | 0·67 | 1·0 | 1·2 | Interval (min) | Author |
|---|---|---|---|---|---|---|---|---|---|
| Cure | WHT An MT | 1·5 | 1·6 | 1·7 | — | 2·1 | — | 30 | Present work |
| Cure | C3H mam Ca | — | — | 1·7–1·8* | — | 1·8 | — | 30 | Sheldon, Foster & Fowler, 1974 |
| Cure | C3H mam Ca | — | — | — | — | 2·3 | — | 30 | Stone & Withers, 1975 |
| Cure | C3H mam Ca | — | — | >1·8 | — | 2·3 | — | 30 | Brown, 1975 |
| Cure | WHT Sq Ca G | — | — | 1·9† | — | 1·8–2·3 | — | 20–30 | Peters, 1976 |
| Cure | WHT Sq Ca D | — | — | — | — | 2·0 | — | 30 | Hill & Fowler, 1977 |
| Regrowth delay | WHT An MT | — | — | — | — | 1·8 | — | 30 | Present work |
| Regrowth delay | CBA Ca NT | — | — | — | — | 2·1 | — | 15 | Denekamp & Harris, 1975 |
| Regrowth delay | CBA Ca F | — | — | — | — | 2·0 | — | 30 | McNally, 1975 |
| Regrowth delay | CBA fast Sa F | — | — | — | — | 1·7 | — | 30 | Begg, 1977‖ |
| Regrowth delay | WHT Sq Ca D | — | — | — | — | 2·2 | — | 30 | Hill & Fowler, 1977 |
| Regrowth delay | WHT bone Sa 2 | — | — | 1·7‡ | — | 1·8 | — | 15–20 | Denekamp & Stewart* |
| Regrowth delay | WHT fibrosarcoma | — | — | — | 1·9 | 1·9 | — | 15–20 | Denekamp & Stewart* |
| $^{125}$IUdR loss | CBA fast Sa F | — | 1·0 | 1·0‡ | — | 1·5 | — | 30 | Begg, 1977‖ |
| Lung colony | C3H Sa KHT | — | — | — | — | — | 1·9§ | 60 | Rauth, Kaufman & Thomson, 1975 |
| Cell dil. *in vitro* | EMT 6 | 2·4 | 2·7 | 2·4 | — | — | 2·9¶ | 30 | Brown, 1975 |
| Cell dil. *in vitro* | CBA Sa F | — | 1·3 | — | — | 2·2 | — | 30 | McNally, 1975 |
| Cell dil. *in vitro* | EMT 6 | — | — | — | — | 2·2 | — | 30 | Bleehen, 1976 |
| Cell dil. *in vitro* | WHT An MT | — | — | — | — | 1·6 | — | 30 | McNally & Sheldon, 1977 |
| Skin clone | — | 1·4 | 1·6 | — | — | 1·9–2·1 | — | 10–20 | Denekamp, Michael & Harris, 1974 |

* Unpublished.

† 0·25 mg/g.

‡ 0·5 mg/g.

§ 1·5 mg/g.

¶ 45 min.

‖ Method uses relatively low X-ray test doses: 10 gray.

TABLE IV.—*Radiosensitization in Mice by Single Doses of Metronidazole*

| Experimental system | Tumour | X-ray dose enhancement for different drug concentrations (mg/g) 0·1 | 0·25 | 0·75 | 1·0 | 1·5 | 2·5 | Interval (min) | Author |
|---|---|---|---|---|---|---|---|---|---|
| Cure | WHT An MT | 1·2 | — | — | 1·5 | — | — | 30 | Present work |
| Cure | C3H mam Ca | — | — | — | — | — | 1·4* | 30 | Begg, Sheldon & Foster, 1974 |
| Cure | C3H mam Ca | 1·2† | — | — | — | — | — | 30 | Stone & Withers, 1974 |
| Cure | WHT Sq Ca G | — | 1·3 | — | — | — | — | 20–30 | Peters, 1976 |
| Regrowth delay | CBA Ca NT | — | — | 1·6 | — | — | — | 15–20 | Denekamp & Harris, 1975 |
| $^{125}$IUdR loss | CBA Sa F | — | — | — | — | 1·3* | — | 30 | Begg, Sheldon & Foster, 1974 |
| Lung colony | C3H Sa KHT | — | — | — | — | 1·5 | — | 15 | Rauth & Kaufman, 1975 |
| Cell dil. *in vitro* | EMT 6 | — | — | — | 1·8 | — | — | 30 | Brown, 1975 |
| Skin clone | — | ~1·3 | 1·3 | — | 1·3 | 1·3* | 1·3–1·5* | 10–20 | Denekamp, Michael & Harris, 1974 |

All drugs given i.p. except: *—orally in tragacanth gum; †—i.v. at 2·5 mg/mouse has been established recently in trials of this drug as a potential tumour radiosensitizer. The maximum tolerated dose is about 180 mg/kg, which produces a serum level of 200 μg/ml (Deutsch *et al.*, 1975). Thus an ER of 1·2 obtained for the lowest dose tested in the anaplastic MT tumour is probably a slight underestimate of what could be achieved clinically, but nevertheless still corresponds to an increase in the local control rate of from 20 to 80% (see Fig. 4). Furthermore, metronidazole has already shown a distinct benefit in the treatment of patients with glioblastoma, using an unconventional fractionation scheme of 9 × 3 gray (Urtasun *et al.*, 1976). The average lifespan of the patients was increased significantly in the metronidazole-treated group.

*Ro-11-3696* has a 5-nitroimidazole ring structure (like metronidazole) with a 2-hydroxy-3-methoxypropyl side chain (like Ro-07-0582). In mammalian cells *in vitro* it has been found to be a better radiosensitizer than metronidazole, but not as good as Ro-07-0582. A maximum ER of 2·4 was obtained for 2150 μg/ml of Ro-11-3696 *in vitro* (Adams *et al.*, 1976).

In the present work, an ER of 1·3 was obtained with 0·33 mg/g of drug. A direct comparison of this response with that obtained *in vitro* is not possible, as no serum measurements have been made in mice.

*Nifurpipone dihydrochloride*, the only nitrofuran tested, is water-soluble, and has been shown specifically to radiosensitize hypoxic mammalian cells *in vitro* by a factor of at least 2·3 (*cf* OER 2·7) (Adams *et al.*, 1974). *In vivo*, the drug does not appear to be so promising. The drug is more toxic than the nitroimidazoles tested, having an $LD_{50}$ in WHT/Ht mice of 0·4 mg/g. The maximum ER that has been observed *in vivo* is 1·45 for artificially hypoxic mouse skin (Denekamp, Michael and Harris, 1974) at a dose of 0·24 mg/g. At the slightly lower dose used here (0·2 mg/g) the ER was only 1·2 (Fig. 3, Table II). Thus the sensitization that can be achieved *in vivo*, with doses that are close to the lethal range, is very much less than would be anticipated from *in vitro* data. The reason for this is not known. Inactivation by binding with serum proteins does not seem to be the reason, since changing the serum concentration *in vitro* did not effect cytotoxicity (Adams *et al.*, 1974). A more likely reason is rapid metabolism or excretion, properties that have limited the usefulness of other nitrofuran compounds (Adams *et al.*, 1974).

*ICRF 159* is the only compound tested that was not selected on the basis of its electron affinity, although its ketone groups do make it weakly electron affinic. It has been reported to have many different actions, including blocking the cell cycle at the radiosensitive G2/M phase, potentiating the action of radiomimetic alkylating agents, and 'normalising' the developing tumour vasculature (so possibly reducing the tumour hypoxia) (Bakowski, 1976).

The effectiveness of ICRF 159 as a radiosensitizer in mice is summarized in Table V. Hellmann and Murkin (1974) achieved an ER of about 2 when 1·25 mg/kg was injected 60 min before each of 5 fractions of X-rays. Similarly, Peters (1976) observed an ER ⩾ 1·2 when mice

TABLE V.—*Radiosensitization of Mouse Tumour Systems by ICRF* 159. *In all Cases the Interval between Final Injection of Drug and Start of Irradiation was* 60 *min*

| Experimental system | Tumour | Treatment | ER | Author |
|---|---|---|---|---|
| Cure | WHT An MT | 30 μg/g before single dose X-rays* | 1·05 | Present work |
| Cure | C3H mam Ca | 30 μg/g before single dose X-rays† | 0·99 | Sheldon, unpub. |
| Cure | WHT Sq Ca G | 30 μg/g before single dose X-rays* | >1·19 | Peters, 1976 |
| Regrowth delay | S 180 | 1·25 μg/g before each of 5 fractions | ~2 | Hellmann & Murkin, 1974 |

* Drug injected daily from implant to day of irradiation.
† Drug injected for only 5 days prior to irradiation.

which had been injected daily, (from implantation to day of irradiation,) with 30 mg/kg, were given the final injection 60 min before a single X-ray dose.

In the present work, we administered 30 mg/kg ICRF 159 in the same manner as Peters (1976), but achieved a disappointing ER of only 1·05. Likewise, with a C3H mammary tumour (unpublished), using 30 mg/kg injected for 5 days before a single X-ray dose, with the final injection 60 min before irradiation, we observed no radiosensitization.

In conclusion, the effectiveness of ICRF 159 as a radiosensitizing agent *in vivo* has differed greatly between tumours, and in the present tumour it is much less effective than the other compounds tested.

### *Effect of radiosensitizing drugs in the development of metastases*

The incidence of metastasis is relatively low for this tumour, comprising only about 5% of the mice treated with X-rays above. None of the drugs significantly increased the incidence of pulmonary metastasis. In 2 experiments, using 1 mg/g of Ro-07-0582 and 0·03 mg/g ICRF 159, no metastases were observed, but the groups of mice were not large enough for this reduction from 5% to 0% to be significant at the 5% level of probability. Nevertheless, the reduction in metastases with ICRF 159 is in agreement with that of other workers who have shown that the drug prevents the seeding of metastases (Hellmann and Burrage, 1969; Salisbury, Burrage and Hellmann, 1970; Peters, 1975). However, in similar $TCD_{50}$ experiments with the C3H mammary carcinoma, which has a higher natural incidence of metastases, we observed no reduction in the level of metastases (8/30 *vs* 5/19) in those mice whose tumours were locally controlled (Sheldon and Hill, unpublished). Since ICRF 159 must be administered from the day of implantation to achieve its antimetastatic effect, and these experiments included administration of 0·03 mg/g/day for only 5 days prior to irradiation, whereas implantation had been performed 2–6 weeks before, it is probable that the metastases had already seeded before the treatment with the drug. However, this particular aspect makes the drug of limited clinical applicability as an antimetastatic agent.

### CONCLUSIONS

(1) In this rapidly growing tumour, 80 days is long enough to wait for assessing the local control of irradiated tumours.

(2) The hypoxic fraction is difficult to determine. The radioresistant tumour ($TCD_{50}$ = 79 gray) is not made more radioresistant by clamping. This is compatible with 100% of the cells being hypoxic. However, the differential ability of chronically hypoxic cells to recover from potentially lethal damage makes this also compatible with 5% of the cells being hypoxic, as estimated by McNally. The change in ER with the size of X-ray dose also suggests that less than 100% of the cells are hypoxic.

(3) No drug tested significantly increased the observed incidence of metastasis.

(4) The most effective radiosensitizer was Ro-07-0582, with metronidazole and Ro-11-3696 next, and then nifurpipone dihydrochloride, with no significant radiosensitization being achieved with ICRF 159.

(5) Ro-07-0582 and metronidazole are nearly as effective radiosensitizers *in vivo* as they are *in vitro*. Ro-11-3696, however, was not as effective *in vivo* as would have been predicted from the *in vitro* studies, and nifurpipone dihydrochloride was very much less effective *in vivo* than *in vitro*.

(6) Ro-11-3696 which is a 5-nitroimidazole like metronidazole but has the same side chains as Ro-07-

0582, was only as effective a radiosensitizer *in vivo* as metronidazole.

We should like to thank Professors J. F. Fowler and R. Oliver and Drs J. Denekamp and H. B. Hewitt for their advice on the preparation of this manuscript; Professor G. E. Adams for encouragement; Mrs E. Marriott for typing; Dr H. B. Hewitt for providing the tumour; Miss A. Marriott and Mrs S. Bull for care of the mice; Miss S. Fairman for help with implanting the tumours; Messrs R. Ransley, B. Bloomfield and M. Cox for construction of the jigs and metal clamps; Dr J. Denekamp, Miss F. A. Stewart and Mr J. L. Foster for permission to quote their unpublished data; and the Cancer Research Campaign for support.

### REFERENCES

ADAMS, G. E. (1973) Chemical Radiosensitisation of Hypoxic Cells. *Br. med. Bull.*, **29**, 48.

ADAMS, G. E., ASQUITH. J. C. & WATTS M. E. (1974) Electron Affinic Sensitisers for Hypoxic Cells Irradiated *In vitro* and *In vivo*. Current Status. In *Advances in Chemical Radiosensitisation* Vienna: I.A.E.A.

ADAMS, G. E., FLOCKHART, I. R., SMITHEN, C. E., STRATFORD, I. J., WARDMAN, P. & WATTS, M. E. (1976) Electron Affinic Sensitisation: VII. A Correlation between Structures, One-electron Reduction Potentials, and Efficiencies of Nitroimidazoles as Hypoxic Cell Radiosensitisers. *Rad. Res.* **67**, 9.

ASQUITH, J. C., FOSTER, J. L., WILLSON, R. L., INGS, R. & MCFADZEAN, J. A. (1974*a*) Metronidazole (" Flagyl "). A Radiosensitiser of Hypoxic Cells. *Br. J. Radiol.*, **47**, 474.

ASQUITH, J. C., WATTS, M. E., PATEL, K., SMITHEN, C. E. & ADAMS, G. E. (1974*b*) Electron Affinic Sensitisation: V. Radiosensitization of Hypoxic Bacteria and Mammalian Cells *In vitro* by some Nitroimidazoles and Nitropyrazoles. *Rad. Res.*, **60**, 108.

BAKOWSKI, M. T. (1976) ICRF 159, ±1,2-di (3,5-Dioxopiperazin-1-yl) propane NSC-129-943; Razoxane. *Cancer Treatment Review*, **3**, 95.

BEGG, A. C. (1977) The Use of 125-Iododeoxyuridine to Measure Hypoxic Cell Radiosensitisation by Ro-07-0582 in a Solid Murine Tumour. *Rad. Res.* (in press).

BEGG, A. C., SHELDON, P. W. & FOSTER, J. L. (1974) Demonstration of Radiosensitisation of Hypoxic Cells in Solid Tumours by Metronidazole. *Br. J. Radiol.*, **47**, 399.

BLEEHEN, N. M. (1976) A Radiotherapist's View of Radiosensitisers. In *Modifications of Radiosensitivity of Biological Systems*. Vienna: I.A.E.A. p. 1.

BROWN, J. M. (1975) Selective Radiosensitisation of the Hypoxic Cells of Mouse Tumours with the Nitroimidazoles, Metronidazole and Ro-07-0582. *Rad. Res.* **64**, 633.

CHAPMAN, J. D., REUVERS, A. P. & BORSA, J. (1973) Effectiveness of Nitrofuran Derivatives in Sensitising Hypoxic Mammalian Cells to X-rays. *Br. J. Radiol.*, **46**, 623.

DENEKAMP, J. & HARRIS, S. R. (1975) Test of Two Electron-affinic Radiosensitizers *in vivo* using Regrowth in an Experimental Carcinoma. *Rad. Res.* **61**, 191.

DENEKAMP, J., MICHAEL, B. D. & HARRIS, S. R. (1974) Hypoxic Cell Radiosensitizers: Comparative Tests of Some Electron Affinic Compounds using Epidermal Cell Survival *In vivo*. *Rad. Res.*, **60**, 119.

DEUTSCH, G., FOSTER, J. L., MCFADZEAN, J. A. & PARNELL, M. (1975) Human Studies with " High Dose " Metronidazole: A Non-toxic Radiosensitizer of Hypoxic Cells. *Br. J. Cancer*, **31**, 75.

DISCHE, S., SAUNDERS, M. I., LEE, M. E., ADAMS, G. E. & FLOCKHART, I. R. (1977) Clinical Testing of the Radiosensitizer Ro-07-0582—Experience with Multiple Doses. *Br. J. Cancer*, **35**, 567.

FOSTER, J. L., FLOCKHART, I. R., DISCHE, S., GRAY, A., LENNOX-SMITH, I. & SMITHEN, C. E. (1975) Serum Concentration Measurements in Man of the Radiosensitizer Ro-07-0582: Some Preliminary Results. *Br. J. Cancer*, **31**, 679.

FOSTER, J. L. & WILLSON, R. L. (1973) Radiosensitisation of Anoxic Cells by Metronidazole. *Br. J. Radiol.*, **46**, 234.

FOWLER, J. F. (1972) Current Aspects of Radiobiology as Applied to Radiotherapy. *Clin. Radiol.*, **23**, 257.

FOWLER, J. F., SHELDON, P. W., BEGG, A. C., HILL, S. A. & SMITH, A. M. (1975) Biological Properties and Response to X-rays of First Generation Transplants of Spontaneous Mammary Carcinomas in C3H Mice. *Int. J. Rad. Biol.*, **27**, 463.

GRAY, A. S., DISCHE, S., ADAMS, G. E., FLOCKHART, I. R. & FOSTER, J. L. (1976) Clinical Testing of the Radiosensitiser Ro-07-0582: I. Dose Tolerance, Serum, and Tumour Concentrations. *Clin. Radiol.*, **27**, 151.

HELLMANN, K. & BURRAGE, K. (1969) Control of Malignant Metastasis by ICRF 159. *Nature, Lond.*, **224**, 273.

HELLMANN, K. & MURKIN, G. E. (1974) Synergism of ICRF 159 and Radiotherapy in Treatment of Experimental Tumours. *Cancer, N.Y.*, **34**, 1033.

HEWITT, H. B., CHAN, D. P. S. & BLAKE, E. R. (1967) Survival Curves for Clonogenic Cells of a Murine Keratinising Squamous Carcinoma Irradiated *In vivo* or under Hypoxic Conditions. *Int. J. Rad. Biol.*, **12**, 535.

HILL, S. A. & FOWLER, J. F. (1977) Radiosensitising and Cytocidal Effects on Hypoxic Cells of Ro-07-0582, and Repair of X-ray Injury, in an Experimental Mouse Tumour. *Br. J. Cancer*, **35**, 461.

MCNALLY, N. J. (1975) The Effect of a Hypoxic Cell Radiosensitiser on Tumour Delay and Cell Survival. Implications for Cell Survival *In situ* and *In vitro*. *Br. J. Cancer*, **32**, 610.

MCNALLY, N. J. & SHELDON, P. W. (1977) The Effect of Radiation on Tumour Growth Delay, Cell Survival and Mouse Cure using the Same Tumour System. *Br. J. Radiol*, (in press).

OLIVARIUS, B. DE F. (1956) Polyneuropathy due to Nitrofurantoin Therapy. *Ugeskr. Laeger*, **118**, 753.

PETERS, L. J. (1975) A Study of the Influence of Various Diagnostic and Therapeutic Procedures applied to a Murine Squamous Carcinoma on its Metastatic Behaviour. *Br. J. Cancer*, **32**, 355.

PETERS, L. J. (1976) Modification of the Radiocurability of a Syngeneic Murine Squamous Carcinoma by its Site of Growth, by Electron-affinic Drugs, and by ICRF 159. *Br. J. Radiol.*, **49**, 708.

RAUTH, A. M. & KAUFMAN, K. (1975) *In vivo* Testing of Hypoxic Radiosensitizers using the KHT Murine Tumour Assayed by the Lung Colony Technique. *Br. J. Radiol.*, **48**, 209.

RAUTH, A. M., KAUFMAN, K. & THOMSON, J. E. (1975) *In vivo* testing of hypoxic cell radiosensitisers. In *Radiation Research, Biomedical, Chemical & Physical Properties*. Ed. O. F. Nygaard, H. I. Adler & W. K. Sinclair. New York: Academic Press.

SALISBURY, A. J., BURRAGE, K. & HELLMANN, K. (1970). Inhibitions of Metastastic Spread by ICRF 159: Selective Deletion of a Malignant Characteristic. *Br. med. J.*, iv, 344.

SCHARER, K. (1972) Selective Alteration of Purkinje Cells in the Dog after Oral Administration of High Doses of Nitromidazole Derivatives. *Verh. dt. Ges. Pathol.*, **56**, 407.

SHELDON, P. W., FOSTER, J. L. & FOWLER, J. F. (1974). Radiosensitisation of C3H Mouse Mammary Tumours by a 2-nitroimidazole Drug. *Br. J. Cancer*, **30**, 560.

SUIT, H. D., SHALEK, R. J. & WETTE, R. (1965) Radiation Response of C3H Mouse Mammary Carcinoma Evaluated in Terms of Cellular Radiation Sensitivity. In *Cellular Radiation Biology*. Baltimore: William & Wilkins Co., p. 574.

STONE, H. B. & WITHERS, H. R. (1974) Tumour and Normal Tissue Response to Metronidazole and Irradiation in Mice. *Radiology*, **113**, 441.

STONE, H. B. & WITHERS, H. R. (1975) Enhancement of the Radioresponse of a Murine Tumour by a Nitroimidazole. *Br. J. Radiol.*, **48**, 411.

THOMLINSON, R. H. (1960) An Experimental Method for Comparing Treatments of Intact Malignant Tumours in Animals and its Application to the Use of Oxygen in Radiotherapy. *Br. J. Cancer*, **14**, 555.

THOMLINSON, R. H., DISCHE, S., GRAY, A. J. & ERRINGTON, L. M. (1976) Clinical Testing of the Radiosensitiser Ro-07-0582: III Response of Tumours. *Clin. Radiol.*, **27**, 167.

THOMLINSON, R. H. & GRAY, L. H. (1955) The Histological Structure of Some Human Lung Cancers and the Possible Implications for Radiotherapy. *Br. J. Cancer*, **9**, 539.

URTASUN, R. C., BAND, P., CHAPMAN, J. D., FELDSTEIN, M. C., MIELKE, B. & FRYER, C. (1976) Radiation and High Dose Metronidazole ("Flagyl®") in Supratentorial Glioblastomas. *New Engl. J. Med.*, **294**, 1364.